# Poly[triaqua­(μ-butane-1,2,3,4-tetra­carboxyl­ato)dicadmium(II)]

**DOI:** 10.1107/S1600536809045255

**Published:** 2009-11-11

**Authors:** Chun-Hong Ma, Yong-Sheng Yan

**Affiliations:** a School of Chemistry and Chemical Engineering, Jiangsu University, Zhenjiang 212013, People’s Republic of China; b College of Chemistry, Jilin Normal University, Siping 136000, People’s Republic of China

## Abstract

The asymmetric unit of the title Cd^II^ coordination polymer, [Cd_2_(C_8_H_6_O_8_)(H_2_O)_3_]_*n*_, contains two crystallographically independent Cd^II^ cations, one-half each of two independent anionic butane-1,2,3,4-tetra­carboxyl­ate units (*L*) and three water mol­ecules. Both anionic units lie on inversion centers. One of the Cd^II^ ions is six-coordinated by four carboxyl­ate O atoms from four *L* anions and two water O atoms in a distorted octa­hedral coordination environment. The other Cd^II^ ion is eight-coordinated by seven carboxyl­ate O atoms from four *L* anions and one water O atom. The anionic units bridge neighboring Cd^II^ centers, forming a three-dimensional framework. O—H⋯O hydrogen-bonding inter­actions between the water mol­ecules and carboxyl­ate O atoms further stabilize the structure.

## Related literature

For coordination polymers with tetra­carboxyl­ate ligands, see: Liu *et al.* (2008 ▶); Yang *et al.* (2008 ▶).
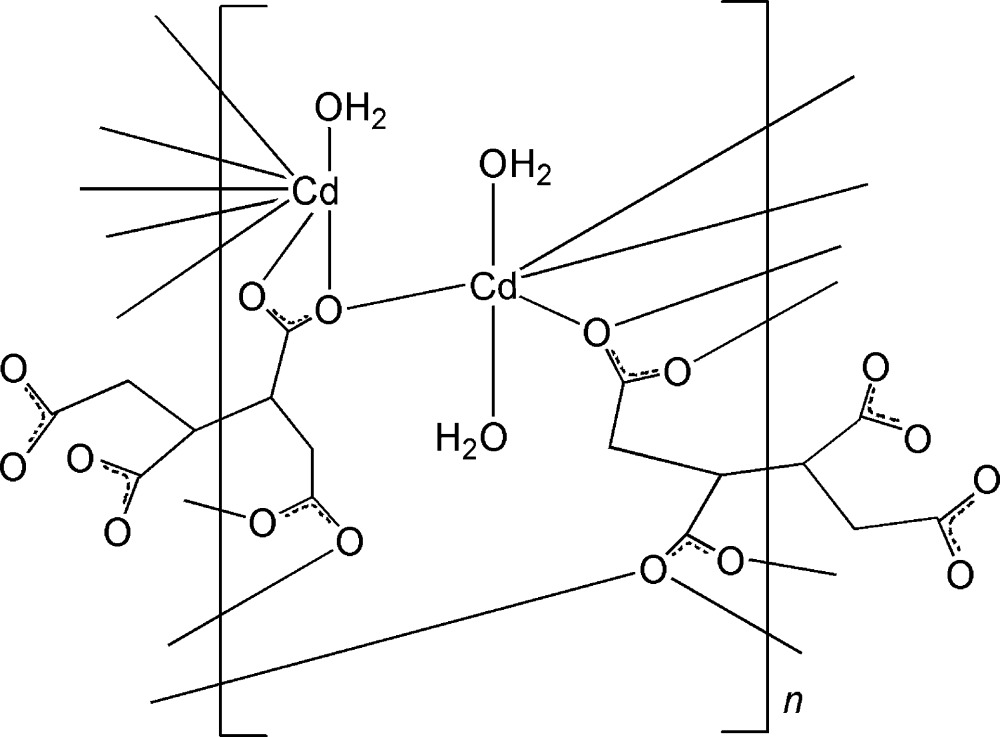


## Experimental

### 

#### Crystal data

[Cd_2_(C_8_H_6_O_8_)(H_2_O)_3_]
*M* *_r_* = 508.98
Triclinic, 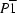

*a* = 7.499 (4) Å
*b* = 7.928 (4) Å
*c* = 11.982 (5) Å
α = 72.886 (4)°
β = 85.748 (4)°
γ = 65.666 (5)°
*V* = 619.4 (6) Å^3^
*Z* = 2
Mo *K*α radiation
μ = 3.49 mm^−1^
*T* = 293 K
0.27 × 0.22 × 0.20 mm

#### Data collection

Bruker APEX CCD area-detector diffractometer
Absorption correction: multi-scan (*SADABS*; Sheldrick, 1996 ▶) *T*_min_ = 0.812, *T*_max_ = 0.910
4846 measured reflections
2847 independent reflections
2225 reflections with *I* > 2σ(*I*)
*R*_int_ = 0.024

#### Refinement

*R*[*F* ^2^ > 2σ(*F* ^2^)] = 0.026
*wR*(*F* ^2^) = 0.053
*S* = 0.92
2847 reflections
208 parameters
4 restraints
H atoms treated by a mixture of independent and constrained refinement
Δρ_max_ = 0.75 e Å^−3^
Δρ_min_ = −0.85 e Å^−3^

### 

Data collection: *SMART* (Bruker, 1997 ▶); cell refinement: *SAINT* (Bruker, 1999 ▶); data reduction: *SAINT*; program(s) used to solve structure: *SHELXS97* (Sheldrick, 2008 ▶); program(s) used to refine structure: *SHELXL97* (Sheldrick, 2008 ▶); molecular graphics: *SHELXTL-Plus* (Sheldrick, 2008 ▶); software used to prepare material for publication: *SHELXL97*.

## Supplementary Material

Crystal structure: contains datablocks global, I. DOI: 10.1107/S1600536809045255/ci2945sup1.cif

Structure factors: contains datablocks I. DOI: 10.1107/S1600536809045255/ci2945Isup2.hkl

Additional supplementary materials:  crystallographic information; 3D view; checkCIF report

## Figures and Tables

**Table 1:** Hydrogen-bond geometry (Å, °)

*D*—H⋯*A*	*D*—H	H⋯*A*	*D*⋯*A*	*D*—H⋯*A*
O1*W*—H*W*11⋯O8^i^	0.81 (5)	2.08 (5)	2.877 (4)	169 (5)
O1*W*—H*W*12⋯O4^ii^	0.85 (5)	2.04 (5)	2.866 (4)	166 (5)
O2*W*—H*W*22⋯O1*W* ^iii^	0.83 (5)	2.00 (5)	2.828 (5)	175 (5)
O2*W*—H*W*21⋯O4^iv^	0.83 (2)	2.02 (2)	2.825 (4)	163 (4)
O3*W*—H*W*31⋯O2^v^	0.84 (2)	1.95 (2)	2.736 (4)	156 (4)
O3*W*—H*W*32⋯O6^vi^	0.85 (2)	2.43 (2)	3.260 (5)	165 (4)

Symmetry codes: (i) 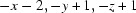; (ii) 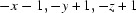; (iii) 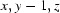; (iv) 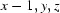; (v) 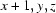; (vi) 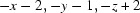.

**Fig. 1.**
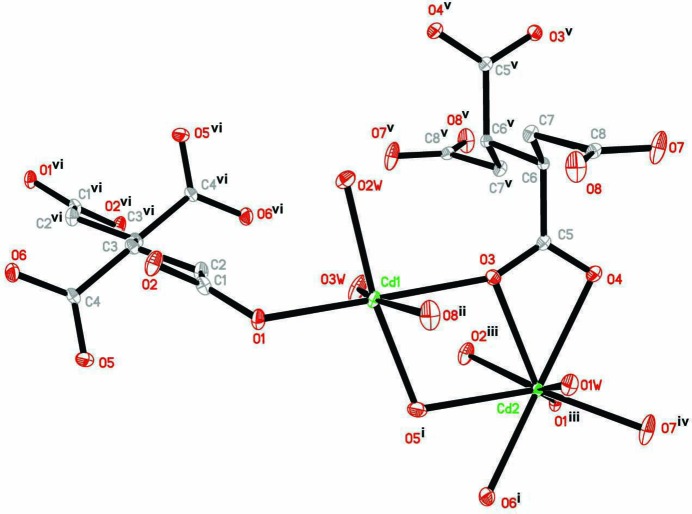
A view of the local coordination of CdII cations in the title compound, showing the atom-numbering scheme. Displacement ellipsoids drawn at the 30% probability level. Symmetry codes: (i) -2-x, -y, 2-z; (ii) -2-x, 1-y, 1-z; (iii) x+1, y, z; (iv) -1-x, 1-y, 1-z); (v) -1-x, -y, 1-z; (vi) -2-x, -1-y, 2-z.

**Fig. 2.**
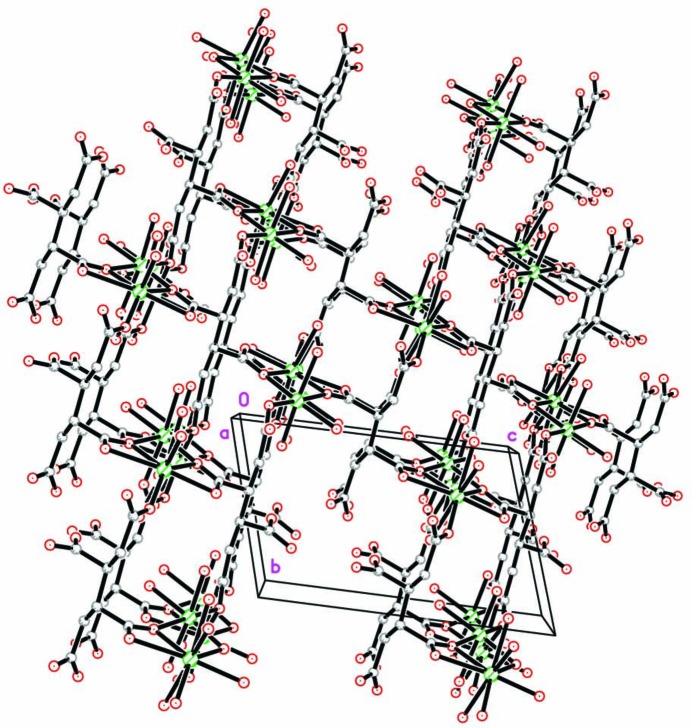
View of the three-dimensional framework structure of the title compound.

## supplementary crystallographic information

### Comment

Over years of intensive studies on tetracarboxylate ligands, transition metals and poly-carboxylates, a vast amount of data have been acquired. As an important family of multidentate O-donor ligands, organic tetracarboxylate ligands, such as 1,2,4,5-benzenetetracarboxylate, have been extensively employed in the preparation of such metal-organic compound (Yang *et al.*, 2008). In this regard, butane-1,2,3,4-tetracarboxylatic acid (H_4_L) is also a good ligand in coordination chemistry due to its strong coordination ability and versatile coordination modes, so much attention has been paid to it in recent years (Liu *et al.*, 2008). In this contribution, H_4_L was selected as a bridging ligand, and a new cadmium coordination polymer, namely [Cd_2_(*L*)(H_2_O)_3_], was obtained.

As shown in Fig. 1, the asymmetric unit of the title Cd^II^ coordination polymer, contains two crystallographically independent Cd^II^ cations, one-half each of two independent *L* anions and three water molecules. The *L* anions lie on inversion centers. One of the Cd^II^ ion, Cd1, is six-coordinated by four carboxylate oxygen atoms from *L* anions and two water oxygen atoms in a distorted octahedral coordination environment. Atom Cd2 is eight-coordinated by seven carboxylate oxygen atoms from *L* anions and one water oxygen atom. The *L* anions bridge neighboring Cd^II^ centers to form a complicated three-dimensional framework structure (Fig. 2). The hydrogen-bonding interactions between water molecules and carboxylate oxygen atoms further stabilize the three-dimensional framework structure of the title compound.

### Experimental

A mixture of CdCl_2_.2H_2_O (0.10 mmol), H_4_L (0.05 mmol) and water (12 ml) was sealed in a Teflon reactor (15 ml), which was heated at 413 K for 3 d and then gradually cooled to room temperature. Purple crystals of the title compound were isolated (yield 68% based on Cd).

### Refinement

H atoms bonded to C atoms were positioned geometrically (C-H = 0.97 or 0.98 Å) and refined as riding, with *U*_iso_(H) = 1.2*U*_eq_(carrier). The water H atoms were located in a difference Fourier map, and were refined with distance restraints of O-H = 0.85 (1) Å and H···H = 1.35 (1) Å; their U_iso_ values were tied to those of parent atoms by a factor of 1.5.

### Crystal data

**Table:**

[Cd_2_(C_8_H_6_O_8_)(H_2_O)_3_]	*Z* = 2
*M**_r_* = 508.98	*F*(000) = 488
Triclinic, *P*1	*D*_x_ = 2.729 Mg m^−^^3^
Hall symbol: -P 1	Mo *K*α radiation, λ = 0.71073 Å
*a* = 7.499 (4) Å	Cell parameters from 2847 reflections
*b* = 7.928 (4) Å	θ = 3.0–29.1°
*c* = 11.982 (5) Å	µ = 3.49 mm^−^^1^
α = 72.886 (4)°	*T* = 293 K
β = 85.748 (4)°	Block, colourless
γ = 65.666 (5)°	0.27 × 0.22 × 0.20 mm
*V* = 619.4 (6) Å^3^	

### Data collection

**Table:**

Bruker APEX CCD area-detector diffractometer	2847 independent reflections
Radiation source: fine-focus sealed tube	2225 reflections with *I* > 2σ(*I*)
graphite	*R*_int_ = 0.024
ω scans	θ_max_ = 29.1°, θ_min_ = 3.0°
Absorption correction: multi-scan (*SADABS*; Sheldrick, 1996)	*h* = −10→9
*T*_min_ = 0.812, *T*_max_ = 0.910	*k* = −9→9
4846 measured reflections	*l* = −16→12

### Refinement

**Table:**

Refinement on *F*^2^	Primary atom site location: structure-invariant direct methods
Least-squares matrix: full	Secondary atom site location: difference Fourier map
*R*[*F*^2^ > 2σ(*F*^2^)] = 0.026	Hydrogen site location: inferred from neighbouring sites
*wR*(*F*^2^) = 0.053	H atoms treated by a mixture of independent and constrained refinement
*S* = 0.92	*w* = 1/[σ^2^(*F*_o_^2^) + (0.024*P*)^2^] where *P* = (*F*_o_^2^ + 2*F*_c_^2^)/3
2847 reflections	(Δ/σ)_max_ = 0.001
208 parameters	Δρ_max_ = 0.75 e Å^−^^3^
4 restraints	Δρ_min_ = −0.85 e Å^−^^3^

### Special details

**Table:**

Geometry. All e.s.d.'s (except the e.s.d. in the dihedral angle between two l.s. planes) are estimated using the full covariance matrix. The cell e.s.d.'s are taken into account individually in the estimation of e.s.d.'s in distances, angles and torsion angles; correlations between e.s.d.'s in cell parameters are only used when they are defined by crystal symmetry. An approximate (isotropic) treatment of cell e.s.d.'s is used for estimating e.s.d.'s involving l.s. planes.
Refinement. Refinement of *F*^2^ against ALL reflections. The weighted *R*-factor *wR* and goodness of fit *S* are based on *F*^2^, conventional *R*-factors *R* are based on *F*, with *F* set to zero for negative *F*^2^. The threshold expression of *F*^2^ > σ(*F*^2^) is used only for calculating *R*-factors(gt) *etc*. and is not relevant to the choice of reflections for refinement. *R*-factors based on *F*^2^ are statistically about twice as large as those based on *F*, and *R*- factors based on ALL data will be even larger.

### Fractional atomic coordinates and isotropic or equivalent isotropic displacement parameters (Å^2^)

**Table:**

	*x*	*y*	*z*	*U*_iso_*/*U*_eq_	
C1	−1.1870 (5)	−0.0435 (5)	0.8775 (3)	0.0222 (8)	
C2	−1.0228 (5)	−0.2392 (5)	0.9250 (4)	0.0242 (9)	
H2A	−0.9498	−0.2348	0.9868	0.029*	
H2B	−0.9344	−0.2675	0.8632	0.029*	
C3	−1.0890 (5)	−0.4024 (5)	0.9729 (3)	0.0195 (8)	
H3	−1.1514	−0.4154	0.9087	0.023*	
C4	−1.2362 (5)	−0.3626 (5)	1.0672 (3)	0.0180 (8)	
C5	−0.5128 (5)	0.1885 (5)	0.5710 (3)	0.0157 (7)	
C6	−0.5234 (5)	0.1089 (5)	0.4728 (3)	0.0138 (7)	
H6	−0.4237	0.1222	0.4175	0.017*	
C7	−0.7242 (5)	0.2187 (5)	0.4086 (3)	0.0198 (8)	
H7A	−0.7313	0.1558	0.3518	0.024*	
H7B	−0.8234	0.2119	0.4643	0.024*	
C8	−0.7700 (5)	0.4286 (5)	0.3463 (3)	0.0186 (8)	
O1	−1.1495 (4)	0.1055 (4)	0.8533 (2)	0.0254 (6)	
O2	−1.3566 (4)	−0.0238 (4)	0.8590 (3)	0.0316 (7)	
O1W	−0.7754 (4)	0.6162 (4)	0.6382 (3)	0.0295 (7)	
HW11	−0.847 (7)	0.565 (7)	0.635 (4)	0.044*	
HW12	−0.721 (7)	0.639 (7)	0.574 (4)	0.044*	
O3	−0.6391 (4)	0.2043 (4)	0.6460 (2)	0.0230 (6)	
O2W	−0.9660 (4)	0.0105 (5)	0.6309 (3)	0.0293 (7)	
HW22	−0.916 (7)	−0.104 (7)	0.631 (4)	0.044*	
HW21	−1.084 (3)	0.058 (6)	0.611 (4)	0.044*	
O4	−0.3718 (4)	0.2352 (4)	0.5789 (2)	0.0249 (6)	
O3W	−0.6465 (4)	−0.1422 (4)	0.8435 (3)	0.0421 (9)	
HW31	−0.545 (5)	−0.136 (6)	0.864 (4)	0.063*	
HW32	−0.628 (7)	−0.259 (4)	0.875 (4)	0.063*	
O5	−1.2174 (4)	−0.2778 (4)	1.1353 (2)	0.0322 (7)	
O6	−1.3702 (4)	−0.4211 (4)	1.0774 (2)	0.0293 (7)	
O7	−0.6349 (4)	0.4662 (4)	0.2971 (3)	0.0450 (9)	
O8	−0.9426 (4)	0.5479 (4)	0.3476 (3)	0.0386 (8)	
Cd1	−0.89424 (4)	0.14635 (4)	0.75659 (2)	0.01890 (8)	
Cd2	−0.48747 (4)	0.31540 (4)	0.76500 (2)	0.01614 (8)	

### Atomic displacement parameters (Å^2^)

**Table:**

	*U*^11^	*U*^22^	*U*^33^	*U*^12^	*U*^13^	*U*^23^
C1	0.019 (2)	0.0184 (19)	0.021 (2)	−0.0033 (16)	0.0030 (16)	−0.0005 (16)
C2	0.0179 (19)	0.0178 (19)	0.032 (2)	−0.0057 (16)	0.0038 (17)	−0.0031 (17)
C3	0.0173 (19)	0.0170 (19)	0.020 (2)	−0.0034 (16)	0.0041 (16)	−0.0056 (16)
C4	0.0171 (18)	0.0115 (17)	0.0177 (19)	−0.0006 (15)	0.0044 (15)	−0.0020 (15)
C5	0.0149 (18)	0.0066 (15)	0.0194 (19)	−0.0002 (14)	−0.0035 (15)	−0.0002 (14)
C6	0.0117 (17)	0.0137 (17)	0.0149 (18)	−0.0045 (14)	0.0010 (14)	−0.0037 (14)
C7	0.0176 (19)	0.0157 (18)	0.022 (2)	−0.0057 (15)	−0.0049 (16)	−0.0001 (15)
C8	0.020 (2)	0.0136 (18)	0.020 (2)	−0.0054 (16)	−0.0048 (16)	−0.0029 (15)
O1	0.0239 (14)	0.0194 (14)	0.0319 (16)	−0.0115 (12)	0.0077 (13)	−0.0038 (12)
O2	0.0195 (15)	0.0229 (15)	0.0472 (19)	−0.0093 (12)	−0.0054 (13)	−0.0002 (14)
O1W	0.0262 (16)	0.0263 (16)	0.0362 (18)	−0.0125 (13)	0.0027 (14)	−0.0068 (14)
O3	0.0205 (14)	0.0247 (14)	0.0276 (15)	−0.0101 (12)	0.0100 (12)	−0.0139 (12)
O2W	0.0237 (15)	0.0300 (16)	0.0406 (18)	−0.0120 (14)	0.0021 (14)	−0.0177 (15)
O4	0.0241 (14)	0.0297 (15)	0.0276 (15)	−0.0160 (13)	0.0017 (12)	−0.0104 (12)
O3W	0.0237 (17)	0.0224 (16)	0.073 (3)	−0.0105 (14)	−0.0142 (16)	0.0028 (16)
O5	0.0320 (16)	0.0449 (18)	0.0316 (17)	−0.0197 (14)	0.0097 (13)	−0.0241 (15)
O6	0.0237 (15)	0.0331 (16)	0.0367 (17)	−0.0155 (13)	0.0118 (13)	−0.0150 (14)
O7	0.0289 (17)	0.0218 (16)	0.075 (3)	−0.0138 (14)	0.0029 (17)	0.0039 (16)
O8	0.0228 (16)	0.0213 (15)	0.052 (2)	0.0002 (13)	0.0038 (15)	0.0030 (14)
Cd1	0.01652 (15)	0.01605 (14)	0.02473 (16)	−0.00788 (11)	0.00187 (12)	−0.00519 (12)
Cd2	0.01470 (14)	0.01407 (14)	0.01976 (15)	−0.00613 (11)	0.00438 (11)	−0.00553 (11)

### Geometric parameters (Å, °)

**Table:**

C1—O2	1.245 (4)	O1W—Cd2	2.601 (3)
C1—O1	1.272 (4)	O1W—HW11	0.81 (5)
C1—C2	1.502 (5)	O1W—HW12	0.85 (5)
C2—C3	1.517 (5)	O3—Cd1	2.370 (3)
C2—H2A	0.97	O3—Cd2	2.430 (3)
C2—H2B	0.97	O2W—Cd1	2.295 (3)
C3—C4	1.528 (5)	O2W—HW22	0.83 (5)
C3—C3^i^	1.560 (7)	O2W—HW21	0.831 (19)
C3—H3	0.98	O4—Cd2	2.495 (3)
C4—O5	1.247 (4)	O3W—Cd1	2.270 (3)
C4—O6	1.254 (4)	O3W—HW31	0.842 (19)
C5—O3	1.251 (4)	O3W—HW32	0.849 (19)
C5—O4	1.274 (4)	O5—Cd1^iv^	2.267 (3)
C5—C6	1.511 (5)	O5—Cd2^iv^	2.521 (3)
C6—C7	1.522 (5)	O6—Cd2^iv^	2.303 (3)
C6—C6^ii^	1.549 (7)	O7—Cd2^v^	2.201 (3)
C6—H6	0.98	O8—Cd1^vi^	2.221 (3)
C7—C8	1.510 (5)	Cd1—O8^vi^	2.221 (3)
C7—H7A	0.97	Cd1—O5^iv^	2.267 (3)
C7—H7B	0.97	Cd2—O7^v^	2.201 (3)
C8—O7	1.232 (5)	Cd2—O6^iv^	2.303 (3)
C8—O8	1.252 (4)	Cd2—O2^vii^	2.381 (3)
O1—Cd1	2.252 (3)	Cd2—O1^vii^	2.490 (3)
O1—Cd2^iii^	2.490 (3)	Cd2—O5^iv^	2.521 (3)
O2—Cd2^iii^	2.381 (3)		
			
O2—C1—O1	119.4 (3)	Cd1—O3W—HW32	139 (3)
O2—C1—C2	121.9 (3)	HW31—O3W—HW32	104 (3)
O1—C1—C2	118.7 (3)	C4—O5—Cd1^iv^	165.6 (2)
C1—C2—C3	114.3 (3)	C4—O5—Cd2^iv^	87.7 (2)
C1—C2—H2A	108.7	Cd1^iv^—O5—Cd2^iv^	105.79 (11)
C3—C2—H2A	108.7	C4—O6—Cd2^iv^	97.8 (2)
C1—C2—H2B	108.7	C8—O7—Cd2^v^	146.3 (3)
C3—C2—H2B	108.7	C8—O8—Cd1^vi^	132.2 (3)
H2A—C2—H2B	107.6	O8^vi^—Cd1—O1	97.13 (10)
C2—C3—C4	111.4 (3)	O8^vi^—Cd1—O5^iv^	83.82 (13)
C2—C3—C3^i^	110.8 (4)	O1—Cd1—O5^iv^	104.20 (10)
C4—C3—C3^i^	108.2 (4)	O8^vi^—Cd1—O3W	161.95 (11)
C2—C3—H3	108.8	O1—Cd1—O3W	100.36 (11)
C4—C3—H3	108.8	O5^iv^—Cd1—O3W	87.57 (13)
C3^i^—C3—H3	108.8	O8^vi^—Cd1—O2W	96.30 (13)
O5—C4—O6	121.1 (3)	O1—Cd1—O2W	84.45 (11)
O5—C4—C3	119.4 (3)	O5^iv^—Cd1—O2W	171.28 (10)
O6—C4—C3	119.5 (3)	O3W—Cd1—O2W	89.84 (13)
O3—C5—O4	119.9 (3)	O8^vi^—Cd1—O3	79.76 (10)
O3—C5—C6	120.3 (3)	O1—Cd1—O3	176.60 (9)
O4—C5—C6	119.8 (3)	O5^iv^—Cd1—O3	76.92 (10)
C5—C6—C7	110.8 (3)	O3W—Cd1—O3	82.86 (11)
C5—C6—C6^ii^	107.5 (3)	O2W—Cd1—O3	94.50 (10)
C7—C6—C6^ii^	111.7 (3)	O7^v^—Cd2—O6^iv^	94.65 (12)
C5—C6—H6	108.9	O7^v^—Cd2—O2^vii^	135.30 (11)
C7—C6—H6	108.9	O6^iv^—Cd2—O2^vii^	98.61 (10)
C6^ii^—C6—H6	108.9	O7^v^—Cd2—O3	127.03 (11)
C8—C7—C6	113.6 (3)	O6^iv^—Cd2—O3	123.75 (9)
C8—C7—H7A	108.8	O2^vii^—Cd2—O3	78.14 (10)
C6—C7—H7A	108.8	O7^v^—Cd2—O1^vii^	82.94 (11)
C8—C7—H7B	108.8	O6^iv^—Cd2—O1^vii^	98.91 (10)
C6—C7—H7B	108.8	O2^vii^—Cd2—O1^vii^	52.95 (8)
H7A—C7—H7B	107.7	O3—Cd2—O1^vii^	119.71 (9)
O7—C8—O8	126.0 (3)	O7^v^—Cd2—O4	84.40 (11)
O7—C8—C7	117.0 (3)	O6^iv^—Cd2—O4	172.55 (9)
O8—C8—C7	116.9 (3)	O2^vii^—Cd2—O4	87.15 (10)
C1—O1—Cd1	127.2 (2)	O3—Cd2—O4	52.67 (8)
C1—O1—Cd2^iii^	90.8 (2)	O1^vii^—Cd2—O4	88.32 (9)
Cd1—O1—Cd2^iii^	118.95 (12)	O7^v^—Cd2—O5^iv^	140.00 (11)
C1—O2—Cd2^iii^	96.6 (2)	O6^iv^—Cd2—O5^iv^	53.43 (9)
Cd2—O1W—HW11	99 (3)	O2^vii^—Cd2—O5^iv^	78.81 (10)
Cd2—O1W—HW12	101 (3)	O3—Cd2—O5^iv^	71.26 (9)
HW11—O1W—HW12	111 (5)	O1^vii^—Cd2—O5^iv^	121.41 (10)
C5—O3—Cd1	157.2 (2)	O4—Cd2—O5^iv^	123.88 (8)
C5—O3—Cd2	95.5 (2)	O7^v^—Cd2—O1W	76.29 (11)
Cd1—O3—Cd2	105.53 (10)	O6^iv^—Cd2—O1W	86.09 (11)
Cd1—O2W—HW22	128 (3)	O2^vii^—Cd2—O1W	146.80 (9)
Cd1—O2W—HW21	114 (3)	O3—Cd2—O1W	72.16 (10)
HW22—O2W—HW21	109 (5)	O1^vii^—Cd2—O1W	158.98 (9)
C5—O4—Cd2	91.9 (2)	O4—Cd2—O1W	86.51 (10)
Cd1—O3W—HW31	116 (3)	O5^iv^—Cd2—O1W	77.91 (11)

Symmetry codes: (i) −*x*−2, −*y*−1, −*z*+2; (ii) −*x*−1, −*y*, −*z*+1; (iii) *x*−1, *y*, *z*; (iv) −*x*−2, −*y*, −*z*+2; (v) −*x*−1, −*y*+1, −*z*+1; (vi) −*x*−2, −*y*+1, −*z*+1; (vii) *x*+1, *y*, *z*.

### Hydrogen-bond geometry (Å, °)

**Table:**

*D*—H···*A*	*D*—H	H···*A*	*D*···*A*	*D*—H···*A*
O1W—HW11···O8^vi^	0.81 (5)	2.08 (5)	2.877 (4)	169 (5)
O1W—HW12···O4^v^	0.85 (5)	2.04 (5)	2.866 (4)	166 (5)
O2W—HW22···O1W^viii^	0.83 (5)	2.00 (5)	2.828 (5)	175 (5)
O2W—HW21···O4^iii^	0.83 (2)	2.02 (2)	2.825 (4)	163 (4)
O3W—HW31···O2^vii^	0.84 (2)	1.95 (2)	2.736 (4)	156 (4)
O3W—HW32···O6^i^	0.85 (2)	2.43 (2)	3.260 (5)	165 (4)

Symmetry codes: (vi) −*x*−2, −*y*+1, −*z*+1; (v) −*x*−1, −*y*+1, −*z*+1; (viii) *x*, *y*−1, *z*; (iii) *x*−1, *y*, *z*; (vii) *x*+1, *y*, *z*; (i) −*x*−2, −*y*−1, −*z*+2.
